# Carbohydrate supplementation and psychophysiological responses during moderate exercise in hypoxia

**DOI:** 10.1186/s12970-019-0331-6

**Published:** 2020-01-06

**Authors:** E. Tavares-Silva, F. F. Donatto, R. M. V. Medeiros, S. A. Santos, A. V. Caris, R. V. Thomatieli-Santos

**Affiliations:** 1 Department of Psychobiology, Universidade Federal de São Paulo, São Paulo, Brazil; 2 Department of Bioscience, Universidade Federal de São Paulo, Rua Silva Jardim, 136 – Vila Mathias, Santos, SP 11015-020 Brazil; 3 Centro Universitário do Rio Grande do Norte (UNI-RN), Natal, Brazil

**Keywords:** Supplementation, Carbohydrate, Hypoxia, Physical exercise, RPE, HR/RPE ratio

## Abstract

**Background:**

Rating of Perceived Exertion (RPE) is a subjective scale to monitor overload and fatigue during exercise. Hypoxia may worsen the perception of fatigue, compromising the self-reported perception of effort and increasing RPE. The objective was to evaluate the effects of carbohydrate (CHO) supplementation on RPE during exercise in hypoxia simulating 4200 m.

**Methods:**

Eight male physically active volunteers performed two exercises at 50% VO_2peak_ and 1% slope: exercise in hypoxia + placebo or exercise in hypoxia + CHO (6% maltodextrin) with supplementation at 20, 40, and 60 min during exercise. Oxygen Saturation (SaO_2_%) was assessed at baseline and after exercise, while RPE and HR were measured each 10 min during the trial.

**Results:**

SaO_2_% decreased after exercise in both conditions of hypoxia compared to rest. The RPE did not differ between groups. However, the RPE increased in hypoxia after 20 min of exercise in relation to 10 min. The Area Under the Curve (AUC) of RPE was lower in hypoxia + CHO compared to hypoxia. The AUC of the HR/RPE ratio in the hypoxia + CHO group was higher in relation to hypoxia.

**Conclusions:**

Our results indicate that CHO supplementation does not change RPE induced by 60 min of exercise at 50% VO_2peak_ in hypoxia equivalent to 4200 m at the different times analyzed. However, in hypoxia + CHO the (AUC)-60 min of total RPE decreased during exercise, while the heart rate/RPE ratio improved, indicating lower RPE in the hypoxic environment.

## Introduction

The Rating of Perceived Exertion (RPE) is a subjective scale used to prescribe the intensity and volume of exercise and measure the level of fatigue after an acute bout of exercise [3, 27]. Several studies have demonstrated a high correlation between the RPE and physiological parameters such as Heart Rate (HR), lactate, and VO_2max_ [10, 12, 15]. In addition, RPE presents psychobiological aspects, including mood state and cognitive and physiological parameters on a single scale [29].

Despite the importance of RPE in normoxic conditions, little is known about the behavior of this scale in hypoxic conditions or high altitudes. Indeed, exposure to hypoxia can worsen cognitive functions [26], affecting mood state variables such as tension, vigor, fatigue, and mental confusion [30, 31] limiting the ability to make decisions [26, 36, 37]. These results suggest worsening of the self-reported perception of effort. One of the few studies that evaluated the effects of hypoxia on the RPE showed that individuals who presented a higher number of symptoms of acute mountain sickness also reported higher levels of perceived exertion [20]. In addition, Souza et al. [30, 31] demonstrated that moderate exercises performed in hypoxic environments for 45 min increase the systolic pressure of young males, as well as modifying the mood state and increasing anxiety [30, 31].

In normoxic environments, different nutritional strategies are commonly used, before, during, and after performing physical exercises, with the ability to induce psycho-physiological modulations, Close et al. [8]. For some time, carbohydrates have received attention in sports nutrition due to their role in performance and adaptability to training, as they provide essential fuel for the brain and central nervous system. Carbohydrates are versatile substrates for muscle work, aiding muscle to withstand exercises in a wide range of intensities due to their use by the anaerobic and oxidative pathways [34].

Specifically regarding RPE, Backhouse et al. [5] demonstrated that carbohydrates could influence RPE in normoxia. However, the influence of carbohydrates in hypoxia environments on RPE and cognition functions is unclear. Golja et al. [14] demonstrate that carbohydrate supplementation in hypoxia causes higher ventilation and oxygen saturation in healthy young males, this being a possible mechanism for the influence of carbohydrates on a lower RPE during physical exercise. Moreover, the importance of carbohydrates during exercise in moderate hypoxia environments is higher in hypoxia than normoxia conditions due to higher endogenous carbohydrate oxidation in this condition [21].

Nevertheless, little is known about the influence of carbohydrates on RPE in hypoxic environments. Thus, we propose to evaluate the effects of carbohydrate supplementation on RPE during exercise in hypoxia, simulating an altitude of 4200 m. We hypothesized that carbohydrate supplementation would attenuate increased RPE during exercise.

## Material and methods

The present study included eight male volunteers, healthy and physically active. The sample characterization is presented in Table 1. The participation of all volunteers was approved by a doctor after a clinical examination, resting electrocardiogram, and stress test. All volunteers signed the consent form.

**Table 1:** Physiological characteristic from voluntaries

Age (years)	24 ± 3	20–29
Weight (kg)	71.21 ± 12.77	54.00–89.80
Height (cm)	173.86 ± 4.56	167–180
BMI	23.59 ± 3.75	17.63–27.70
VO_2peak_ absolute (l/ml)	3.27 ± 0.40	2.95–4.05
VO_2peak_ relative (mg/kg/min)	46.39 ± 5.62	38.13–54.00
Velocity maximal (km/h)	16.00 ± 1.26	15–18
Time (min)	13.37 ± 1.41	11.40–15.00

*n* = 8 voluntaries

### Experimental design

This is a cross-over study, in which the volunteers visited the laboratory three times. On the first visit, the volunteers carried out the resting and effort electrocardiogram and, simultaneously, the cardiopulmonary exercise test for peak oxygen uptake (VO_2peak_) determination. On the two subsequent visits, the volunteers performed: (I) exercise in hypoxia and placebo supplementation and (II) exercise in hypoxia and carbohydrate supplementation. All procedures were double-blind and randomized with respect to supplementation. There was a 7-day interval between each visit [9].

### Physiological parameters

VO_2peak_ was determined in normoxia using an incremental exercise test on a treadmill (LifeFitness® - 9700HR). The initial velocity was set at 6.0 km/ h, increased by 1.0 km/h per minute until voluntary exhaustion. Respiratory and metabolic variables were obtained breath by breath using a metabolic system (Cosmed PFT4, Rome, Italy). A 1% slope on the treadmill was maintained throughout the test.

The volunteers performed 60 min of acute exercise at 50% VO_2peak_, and a 1% slope on the treadmill in the hypoxia condition simulated to 4200 m. All the physical exercise sessions were performed after fasting for 3 hours, to avoid possible dietary influences, and began at 02:00 pm. The pre-test meal was not controlled, but it was suggested that volunteers eat a light meal, and water intake in the hours preceding the test was ad libitum. The volunteers were advised not to perform strenuous exercises in the 24 h preceding the exercise.

### Carbohydrate supplementation

Volunteers received a 200 ml solution of carbohydrate - CHO (maltodextrina strawberry-flavored) at 6% (w/v), at 20, 40, and 60-min during exercise with 228 kcal, or a placebo 0 kcal (strawberry-flavored Crystal Light® - Kraft Foods, Northfield, IL – USA). The groups received the same volume of placebo or carbohydrate in a double-blind manner.

### Hypoxic environment

The study was performed in a chamber (normobaric chamber; Colorado Altitude Training/12 CAT-Air Unit) for altitude simulations of up to 4200 m, which is equivalent to a barometric pressure of 433 mmHg and fraction of inspired oxygen (FiO2) of 13.5% O_2_. This equipment has two air units allocated on the outside, which allow gas exchange (nitrogen increase and O_2_ reduction). A display inside the chamber shows the simulated altitude in real-time, measured by a module that contains an O_2_ cell sensitive to O_2_ variations.

### Rating of perceived exertion determination

The RPE scale was used as a measure of perceived exertion during exercise. The scale ranges from 6 to 20, with anchors ranging from “very, very light” to “very, very hard” [7]. Heart rate (HR) was measured using the Frequency meter (Polar®, Advantage Model NV, Kempele, Finland), and Hemoglobin O_2_ saturation (SaO_2_%) was measured by a finger oximeter (FingerPulse® model MD300C202, Minnesota - USA). SaO_2_% was assessed at baseline and after exercise, while RPE and HR were measured each 10 min during the trial.

### Statistical analysis

Results of SaO_2_%, HR, RPE, and the HR/RPE ratio are expressed as mean standard ± deviation, and statistical analyses were performed by two-way repeated-measures ANOVA, after the Shapiro-Wilk normality test, followed by the Tukey post hoc test, at *p* < 0.05. The Area Under the Curve (AUC) was calculated using the trapezoidal rule to quantify the overall response of RPE and HR/RPE to exercise in the two different conditions studied. Statistical analyses were performed using UNIANOVA.

## Results

**Fig. 1:**
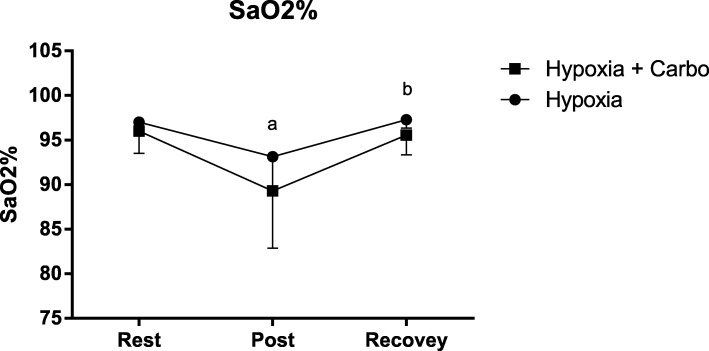
Hemoglobin Saturation. SaO_2_% in hypoxia and hypoxia + CHO conditions for *n* = 8 volunteers. The results represent the mean ± SD. (**a**) Different from Rest (**b**) different from Post in Hypoxia and Hypoxia + CHO group

There was decrease in SaO_2_% after exercise in hypoxia (92.37 ± 3.50, F(1,6) = 3,79; *p* = 0.05) and hypoxia + CHO (89.25 ± 5.94, F(1,6) = 9,48; *p* = 0.03) in relation to rest in both groups (97.00 ± 0.92 / 96.00 ± 2.32), as shown in Fig. 1. In relation to HR there were increases at all moments during exercise in comparison to baseline, similarly in hypoxia F(1,6) = 16.64; *p* = 0.001 and hypoxia + CHO F(1,6) = 18.27; p = 0.001 as demonstrated in Fig. 2.1(b). The results of RPE are demonstrated in Fig. 2.2 (b). In hypoxia there was an increase at 20 min (12.37 ± 1.30 F(1,6) = 3.59; *p* = 0.041) in relation to 10 min (10.5 ± 1.30). In hypoxia + CHO supplementation we did not observe any increase at 20 (10.75 ± 1.75), 30 (11.50 ± 2.07), 40 (11.87 ± 2.35), 50 (12.87 ± 4.54), and 60 (11.87 ± 1.95) minutes in relation to 10 (9.62 ± 1.59) minutes F(1,6) = 2,03; p = 0,15). The HR/RPE ratio is shown in Fig. 2.3 (b). No differences were observed between the groups. However, in hypoxia + CHO there was a decrease at 20 min (13.56 ± 1.76) compared to 30 min (12.41 ± 1.46) F(1,6) = 5,51; *p* = 0.01. The AUC of HR did not differ between groups (0.58 ± 0.05 / 0.55 ± 0.04 F(1,5) = 1.69; *p* = 0.20), as shown in Fig. 2.1 (a). The AUC of RPE was lower in hypoxia + CHO (0.47 ± 0.05) compared to hypoxia (0.62 ± 0.07) F(1,4) = 11.66; *p* = 0.007, Fig. 2.2 (a). The AUC of HR/RPE in hypoxia + CHO (0.52 ± 0.02) was significantly higher than in the hypoxia condition (0.47 ± 0.02) F(1,4) = 5.09; *p* = 0.04, Fig. 2.3 (b).

**Fig. 2:**
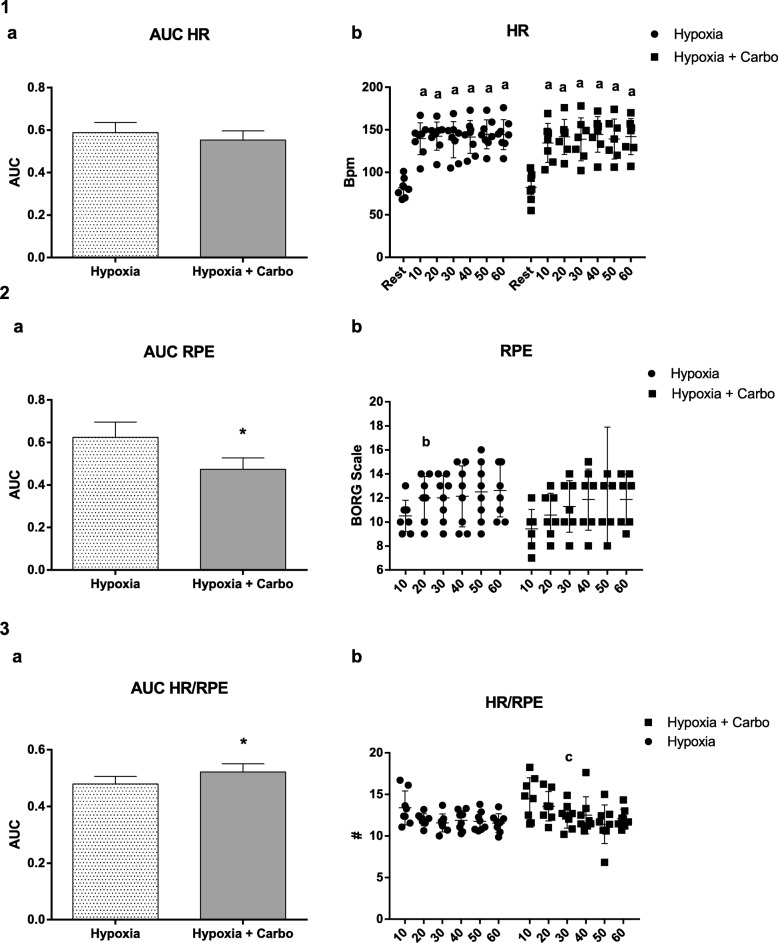
HR and RPE. AUC – HR, RPE and HR/RPE in hypoxia and hypoxia + CHO conditions for n = 8 volunteers. 1(**a**) represents the AUC of Heart Rate, and 1(**b**) the Heart Rate during the different minutes. 2(**a**) represents the AUC of Rating Perception of Exertion, and 2(**b**) the Rating Perception of Exertion during the different minutes. 3(**a**) represents the AUC of the ratio of Heart Rate/Rating Perception Exertion, and 3(**b**) corresponds to the ratio of Heart Rate/Rating Perception Exertion during the different minutes. The results represent the mean ± SD. * different from Hypoxia, (**a**) different from Rest; (**b**) different from 10 min at Hypoxia Group; (**c**) different from 20 min in Hypoxia + CHO group

## Discussion

The RPE represents psychobiological and physiological signs and symptoms on a single scale to evaluate the intensity and feeling of fatigue during exercise. However, little is known about this scale in hypoxia. Thus, the aim of the study was to evaluate the effects of carbohydrate supplementation on RPE during exercise in hypoxia, simulating an altitude of 4200 m. Our results indicate that there was no difference in the RPE during the different times analyzed after 60 min of exercise at moderate intensity in hypoxia. However, carbohydrate supplementation decreased total RPE during exercise in hypoxia, as indicated by a decrease in AUC of RPE and by the AUC of the HR/RPE ratio. The Area Under the Curve is a mathematical formula used to incorporate several time points to detect associations between repeated measures [25]. An extensive review of AUC by Tilaki [35] demonstrated that the analyses of AUC is of great importance for diagnostics, even being used in clinical epidemiology for the diagnosis of biomarkers and classification of disease. Different studies have used this statistical methodology to perform a global analysis of the results rather than just punctual analysis, as verified in the studies of [1, 28, 32].

It is known that during hypoxia exposure, muscle carbohydrate metabolism changes, and there is higher carbohydrate oxidation compared to the normoxia condition. The energy supply via the glycolytic system is enhanced during endurance exercise in hypoxia, lactate and hydrogen ions (H+) are produced by the working muscle via the augmented energy supply from the glycolytic system and subsequently released into the blood circulation by Na+/H+ exchanger isoform 1 and monocarboxylate transporters, which elicits metabolic acidosis (lower muscle pH) [33]. However, exogenous glucose uptake is compromised under hypoxic conditions, demonstrating the severity of exposure to high altitude. In a recent work, O'Hara et al. [23] compared the co-ingestion of glucose and fructose on exogenous and endogenous substrate oxidation during prolonged exercise at high altitude (HA) versus sea level, in women, and the results showed that the rates of exogenous carbohydrate oxidation were significantly lower at HA.

In the present study, we evaluated the SaO_2_% in hemoglobin, as demonstrated in Fig. 1 (a). We observed a significant decrease after exercise in both the hypoxia and hypoxia + CHO conditions, confirming previous studies [6, 22]. A decrease in O_2_ saturation is one of the first consequences of hypoxia, occurring within a few minutes of exposure [19]. Thus, the reduction in SaO_2_% after exercise found in hypoxia confirms the efficiency of the model studied to induce hypoxia and indicates that 4200 m, even for 60 min, is capable of reducing the O_2_ supply to several tissues.

This result could have a significant impact on the RPE since the supply of O_2_ is essential for the preservation of cognitive function and mood. Li et al. [18] showed that mood, including stress, fatigue, and force progressively worsen proportionally to increases in hypoxia. Furthermore, studies have shown that hypoxia can worsen cognitive functions, including memory, learning, attention, and decision making [13, 16, 24, 36].

In the current study, there was an increase in RPE at the 20th min of exercise compared to the 10th minute only in the hypoxia group. In addition, the AUC of RPE was significantly lower when the volunteers were supplemented with carbohydrates. Backhouse et al. [5] demonstrated a reduction in self-reported perception of volitional fatigue in exercise lasting 90 min in normoxia. Furthermore, Fulco et al. [11] did not find a difference in the RPE during exercise in hypoxia, similar to 4300 m after several days of energy deficit, unlike our study. Moreover, RPE increases when there is a decrease in blood glucose, since glucose is the primary fuel for the brain [4].

RPE is influenced by the intensity and volume of exercise and presents high correlations with various physiological measures, including HR [10, 15]. Despite the increase in HR from the 10th min of exercise, there was no difference between the two conditions studied. Fulco et al. [11] found a higher HR after carbohydrate supplementation during exercise in hypoxia as opposed to our results. However, those authors used a different protocol and time of exposure. On the other hand, Ando et al. [2] and Kubota et al. [17] demonstrated differences in HR in a single bout of exercise performed in a hypoxia condition compared to normoxia.

The AUC of RPE demonstrated that carbohydrate supplementation was effective for reducing the effects of simulated hypoxic environments. The explanation is related to a possible increase in ventilation, as demonstrated in the study of Golja et al. [14] and the increase in nutrients due to supplementation during the exercise, decreasing the fatigue generated by physical exercise Backhouse et al. [5].

The HR/RPE ratio may be an index that represents the relationship between a physiological and psychological marker for recording the intensity of the exercise, and the level of fatigue [29]. A reducing HR/RPE suggests an increased feeling of fatigue despite the intensity of the exercise. However, the effect of exercise in hypoxia on the HR/RPE ratio is unknown. In our study, there was no difference in the HR/RPE ratio at the different times analyzed during 60 min of exercise in hypoxia.

On the other hand, the HR/RPE was higher at 20 min in those supplemented with carbohydrate, but not significantly compared to hypoxia. Subsequently, after 30 min, the HR/RPE decreased, indicating that supplementation was efficient for partially preventing the first feelings of fatigue during exercise in hypoxia. Our results are confirmed by the AUC, demonstrating the global decline caused by exercise in hypoxia and recovery due to carbohydrate supplementation. This information on the response to carbohydrate supplementation during exercise can help sports nutritionists target better dietary strategies for athletes under hypoxic conditions.

## Conclusion

In conclusion, our results indicate that carbohydrate supplementation does not change RPE at the different times analyzed during 60 min of exercise at 50% VO_2peak_ in hypoxia equivalent to 4200 m. However, even during 60 min of acute hypoxia, the carbohydrate supplementation significantly decreased the Area Under the Curve (AUC)-60 min during exercise in hypoxia of RPE and improved the HR/RPE ratio, demonstrating the importance of carbohydrates to attenuate the impacts of hypoxic environments.

## Data Availability

The datasets used and/or analyzed during the current study are available from the corresponding author on reasonable request.

## Abbreviations

AUC: Area Under the Curve
CEP: Comitê de Ética e Pesquisa (Ethics Committee for Research)
CHO: Carbohydrate
FiO2: Fraction of inspired oxygen
HA: High Altitude
HR: Heart Rate
O_2_: Oxygen
RPE: Rating of Perceived Exertion
SaO_2_%: Hemoglobin O_2_ saturation
VO_2max_: Maximum oxygen volume
VO_2peak_: Peak oxygen volume

## References

[1] Alsamir Tibana R, Manuel Frade de Sousa N, Prestes J (2019). Is perceived exertion a useful indicator of the metabolic and cardiovascular responses to a metabolic conditioning session of functional fitness?. Sports (Basel).

[2] Ando S, Hatamoto Y, Sudo M, Kiyonaga A, Tanaka H, Higaki Y (2013). The effects of exercise under hypoxia on cognitive function. PLoS One.

[3] Astorino TA, Allen RP, Roberson DW, Jurancich M, Lewis R, McCarthy K (2012). Attenuated RPE and leg pain in response to short-term high-intensity interval training. Physiol Behav.

[4] Astorino TA, Roupoli LR, Valdivieso BR (2012). Caffeine does not alter RPE or pain perception during intense exercise in active women. Appetite.

[5] Backhouse SH, Ali A, Biddle SJ, Williams C (2007). Carbohydrate ingestion during prolonged high-intensity intermittent exercise: impact on affect and perceived exertion. Scand J Med Sci Sports.

[6] Billat VL, Lepretre PM, Heubert RP, Koralsztein JP, Gazeau FP (2003). Influence of acute moderate hypoxia on time to exhaustion at vVO2max in unacclimatized runners. Int J Sports Med.

[7] Borg GAV (1982). Psychophysical bases of perceived exertion. Med Sci Sports Exerc.

[8] Close GL, Hamilton L, Philp A, Burke L, Morton JP (2016). New strategies in sport nutrition to increase exercise performance. Free Radic Biol Med.

[9] Coppel J, Hennis P, Gilbert-Kawai E, Grocott MP (2015). The physiological effects of hypobaric hypoxia versus normobaric hypoxia: A systematic review of crossover trials. Extreme Physiol Med.

[10] Coutts AJ, Rampinini E, Marcora SM, Castagna C, Impellizzeri FM (2009). Heart rate and blood lactate correlates of perceived exertion during small-sided soccer games. J Sci Med Sport.

[11] Fulco CS, Kambis KW, Friedlander AL, Rock PB, Muza SR, Cymerman A (2005). Carbohydrate supplementation improves time-trial cycle performance during energy deficit at 4,300-m altitude. J Appl Physiol.

[12] Garber CE, Blissmer B, Deschenes MR, Franklin BA, Lamonte MJ, Lee IM, Nieman DC, Swain DP (2011). Quantity and quality of exercise for developing and maintaining cardiorespiratory, musculoskeletal, and neuromotor fitness in apparently healthy adults: guidance for prescribing exercise. Med Sci Sports Exerc.

[13] Gibson GE, Pulsinelli W, Blass JP, Duffy TE (1981). Brain dysfunction in mild to moderate hypoxia. Am J Med.

[14] Golja P, Flander P, Klemenc M, Maver J, Princi T (2008). Carbohydrate ingestion improves oxygen delivery in acute hypoxia. High Alt Med Biol.

[15] Haddad M, Chaouachi A, Wong del P, Castagna C, Hue O, Impellizzeri FM, Chamari K (2014). Influence of exercise intensity and duration on perceived exertion in adolescent taekwondo athletes. Eur J Sport Sci.

[16] Hornbein TF, Townes BD, Schoene RB, Sutton JR, Houston CS (1989). The cost the central nervous system of climbing to extremely high altitude. N Engl J Med.

[17] Kubota Y, Fukusaki C, Okaneya S, Maegawa T, Narita K (2015). Effects of short hypoxic pre-exposure on physiological responses to subsequent hypoxic exercise. J Phys Fit Sports Med.

[18] Li XY, Wu XY, Fu C, Shen XF, Wu YH, Wang T. Effects of acute mild and moderate hypoxia on human mood state. Space Med Med Eng. 2000;(1):131–5. 12212624

[19] Mazzeo RS (2008). Physiological responses to exercise at altitude: an update. Sports Med.

[20] Mellor AJ, Woods DR, O’Hara J (2014). Rating of perceived exertion and acute mountain sickness during a high-altitude trek. Aviat Space Environ Med.

[21] Morishima T, Mori A, Sasaki H, Goto K (2014). Impact of exercise and moderate hypoxia on glycemic regulation and substrate oxidation pattern. PLoS One.

[22] Mounier R, Pialoux V, Schmitt L, Richalet JP, Robach P, Coudert J, Clottes E, Fellmann N (2009). Effects of acute hypoxia tests on blood markers in high-level endurance athletes. Eur J Appl Physiol.

[23] O'Hara JP, Duckworth L, Black A, Woods DR, Mellor A, boos C, Gallagher L, Tsakirides C, Arjomandkhah NC, Morrison DJ, Preston T, King RF (2019). Fuel use during exercise at altitude in women with glucose-fructose ingestion. Med Sci Sports Exerc.

[24] Paintal SA (2004). Cognitive functions in extraordinary environments. Indian J Med Res.

[25] Pruessner JC, Kirschbaum C, Meinlschmid G, Hellhammer DH (2003). Two formulas for computation of the area under the curve represent measures of total hormone concentration versus time-dependent change. Psychoneuroendocrinology.

[26] Roach EB, Bleiberg J, Lathan CE (2014). AltitudeOmics: decreased reaction time after high altitude cognitive testing is a sensitive metric of hypoxic impairment. Neuroreport.

[27] Rose EA, Parfitt G (2008). Can the feeling scale be used to regulate exercise intensity?. Med Sci Sports Exerc.

[28] Sakaguchi K, Takeda K, Maeda M (2015). Glucose area under the curve during oral glucose tolerance test as an index of glucose intolerance. Diabetol Int.

[29] Snyder AC, Jeukendrup AE, Hesselink MK, Kuipers H, Foster C (1993). A physiological/psychological indicator of over-reaching during intensive training. Int J Sports Med.

[30] Souza JFT, Giampá SQC, LEMOS VA, De Mello MT, Santos RVT, Antunes HKM (2015). A condição de altitude simulada piora o estado de humor e aumenta a pressão arterial sistólica de jovens saudáveis. Motricidade.

[31] Souza JFT, Giampá SQC, LEMOS VA, Santos SA, Silva ET, De Mello MT, Santos RVT, Antunes HKM (2015). Exercise performed at hypoxia influences mood state and anxiety symptoms. Motriz.

[32] Steiner JL, A Curmaci A, Patrie JT, Gaesser GA, Weltman A (2009). Effects of carbohydrate supplementation on the RPE-blood lactate relationship. Med Sci Sports Exerc.

[33] Sumi D, Kojima C, Goto K (2018). Impact of endurance exercise in hypoxia on muscle damage, inflammatory and performance responses. J Strength Cond Res.

[34] Thomas DT, Erdman KA, Burke LM (2016). American College of Sports Medicine position stand nutrition and athletic performance. Med Sci Sports Exerc.

[35] Tilaki KH (2013). Receiver operating characteristic (ROC) curve analysis for medical diagnostic test evaluation. Caspian J Intern Med.

[36] Virués-Ortega J, Buela-Casal G, Garrido E, Alcázar B (2004). Neuropsychological functioning associated with high-altitude exposure. Neuropsychol Rev.

[37] Zhang G, Zhou SM, Yuan C, Tian HJ, Li P, Gao YQ (2013). The effects of short-term and long-term exposure to a high-altitude hypoxic environment on neurobehavioral function. High Alt Med Biol.

